# BAIAP2L2 Inactivation Does Not Affect Stereocilia Development or Maintenance in Vestibular Hair Cells

**DOI:** 10.3389/fnmol.2022.829204

**Published:** 2022-02-15

**Authors:** Keji Yan, Chengli Qu, Yanfei Wang, Wen Zong, Zhigang Xu

**Affiliations:** ^1^Shandong Provincial Key Laboratory of Animal Cell and Developmental Biology, School of Life Sciences, Shandong University, Qingdao, China; ^2^State Key Laboratory of Microbial Technology, Shandong University, Qingdao, China; ^3^Shandong Provincial Collaborative Innovation Center of Cell Biology, Shandong Normal University, Jinan, China

**Keywords:** inner ear, vestibular hair cells, stereocilia, BAIAP2L2, CAPZB2

## Abstract

Hair cells are mechanosensitive cells in the inner ear, characterized by dozens to hundreds of actin-based stereocilia and one tubulin-based kinocilium on the apical surface of each cell. Two types of hair cells, namely cochlear hair cells and vestibular hair cells (VHCs), are responsible for the sensation of sound and balancing information, respectively. In each hair cell, the stereocilia are organized into rows of increasing heights with the mechano-electrical transduction (MET) channels localized at the tips of shorter-row stereocilia. A so-called “row 2 protein complex” also localizes at the tips of shorter-row mechanotransducing stereocilia, which plays important roles in the maintenance of mechanotransducing stereocilia. Recently, we and others identified BAIAP2L2 as a new component of row 2 complex. *Baiap2l2* inactivation causes degeneration of the mechanotransducing stereocilia in cochlear hair cells, and leads to profound hearing loss in mice. In the present work, we examined the role of BAIAP2L2 in the VHC stereocilia. Confocal microscopy reveals that BAIAP2L2 immunoreactivity is localized at the tips of shorter-row stereocilia in VHCs. However, stereocilia development and maintenance are unaffected in *Baiap2l2^–/–^* VHCs. Meanwhile, MET function of VHCs as well as vestibular functions are also unaffected in *Baiap2l2^–/–^* mice. Further investigations show that the stereociliary tip localization of CAPZB2, another known row 2 complex component, is not affected in *Baiap2l2^–/–^* VHCs, consistent with the unaltered stereocilia morphology. Taken together, our present data show that BAIAP2L2 inactivation does not affect vestibular hair cell stereocilia.

## Introduction

Hair cells are the mechanosensory cells in the inner ear, responsible for converting the mechanical signals into electrical signals, a process referred to as mechano-electrical transduction (MET). Each hair cell harbors dozens to hundreds of actin-based stereocilia and one microtubule-based kinocilium at the apical surface, collectively named hair bundle (Flock and Cheung, 1977). The stereocilia play a pivotal role in MET, whereas the kinocilium is important for hair bundle development (Hudspeth and Jacobs, 1979; Jones et al., 2008).

There are two different types of hair cells in the mammalian inner ear, namely cochlear hair cells and vestibular hair cells (VHCs), which are responsible for the sensation of sound and balancing information, respectively. In each mammalian cochlear hair cell, stereocilia are organized into three rows of increasing heights, forming a characteristic staircase-like pattern (Tilney et al., 1980). Similarly, the stereocilia in mammalian VHCs form a staircase-like pattern with more rows of increasing heights (Krey and Barr-Gillespie, 2019). The MET channels are localized at the tips of shorter-row stereocilia, which are therefore referred to as mechanotransducing stereocilia (Beurg et al., 2009). In either type of hair cells, the development and maintenance of stereocilia is tightly regulated, and several proteins have been identified to play important roles in regulating stereocilia length (Barr-Gillespie, 2015; McGrath et al., 2017; Krey and Barr-Gillespie, 2019; Velez-Ortega and Frolenkov, 2019).

At the tips of the tallest-row stereocilia, there is a so-called “row 1 protein complex” that controls the identity and development of the tallest-row stereocilia (Tadenev et al., 2019; Krey et al., 2020). Meanwhile, at the tips of shorter-row mechanotransducing stereocilia resides a “row 2 protein complex”, which consists of MYO15A-L, EPS8L2, TWF2, and CAPZB2 (Peng et al., 2009; Rzadzinska et al., 2009; Furness et al., 2013; Fang et al., 2015; Avenarius et al., 2017). Evidences suggest that deficiency of row 2 complex components leads to degeneration of the mechanotransducing stereocilia (Furness et al., 2013; Fang et al., 2015). Recently, we and others identified BAI1-associated protein 2-like 2 (BAIAP2L2, also known as Pinkbar) as a new component of row 2 complex (Carlton et al., 2021; Yan et al., 2021). BAIAP2L2 inactivation causes degeneration of the mechanotransducing stereocilia in cochlear hair cells, and leads to profound hearing loss in mice (Carlton et al., 2021; Yan et al., 2021). Furthermore, the stereociliary tip localization of the known row 2 complex component, CAPZB2, is abolished in *Baiap2l2* knockout mice, suggesting that BAIAP2L2 is indispensable for the formation of row 2 complex in cochlear hair cells (Yan et al., 2021).

In the present work, we further investigate the role of BAIAP2L2 in VHC stereocilia. Surprisingly, our results show that albeit localizing at the tips of shorter-row VHC stereocilia, BAIAP2L2 is not required for the development or maintenance of VHC stereocilia, which is in sharp contrast to the results observed in cochlear hair cells.

## Materials and Methods

### Mice

Animal experiments were approved by the Animal Ethics Committee of Shandong University School of Life Sciences (Permit Number: SYDWLL-2020-31) and performed accordingly. *Baiap2l2* and *Lhfpl5* knockout mice were established and maintained as previously reported (Xiong et al., 2012; Yan et al., 2021).

### Whole-Mount Immunostaining

Utricles and saccules were dissected out and fixed with 4% paraformaldehyde (PFA) in PBS for 20 min, followed by permeabilization and blocking with PBT1 (0.1% Triton X-100, 1% BSA, and 5% heat-inactivated goat serum in PBS, pH 7.3) for 40 min. Afterwards, the samples were incubated with primary antibody in PBT1 overnight at 4°C, followed by incubation with corresponding secondary antibody in PBT2 (0.1% Triton X-100 and 0.1% BSA in PBS) for 2 h. After incubation with TRITC-conjugated phalloidin (Sigma-Aldrich, Cat. No. P1951) in PBS for 30 min, the samples were mounted in PBS/glycerol (1:1) and imaged using a confocal microscope with a 1.4NA/63 × Kort M27 objective lens (LSM 900, Zeiss, Germany). The antibodies used in the present study are as follows: rabbit anti-BAIAP2L2 antibody (Sigma-Aldrich, Cat. No. HPA003043); rabbit anti-CAPZB2 antibody (Merck, Cat. No. AB6017); mouse anti-EPS8 antibody (BD Biosciences, Cat. No. 610143); Alexa Fluor 488-conjugated donkey anti-rabbit IgG (Thermo Fisher Scientific, Cat. No. A21206); Alexa Fluor 488-conjugated donkey anti-mouse IgG (Thermo Fisher Scientific, Cat. No. A21202).

### Scanning Electron Microscopy

Mouse temporal bone was dissected out and fixed with 2.5% glutaraldehyde in 0.1 M phosphate buffer overnight at 4°C. Then the utricle and saccule were taken out of the temporal bone and post-fixed with 1% osmium tetroxide in 0.1 M phosphate buffer at 4°C for 2 h. After dehydration in ethanol and critically point drying using a Leica EM CPD300 (Leica, Germany), samples were mounted and sputter coated with platinum (15 nm) using a Cressington 108 sputter coater (Cressington, United Kingdom). Images were taken using a Quanta250 field-emission scanning electron microscope (FEI, Netherlands).

### FM1-43FX Uptake

The sensory epithelia of utricle and saccule were dissected out and incubated with 3 μM FM 1-43FX (Thermo Fisher, Cat. No. F35355) in PBS for 40 s, then fixed with 4% PFA at room temperature for 20 min. After mounting in PBS-glycerol (1:1), the samples were imaged using a confocal microscope with a 0.8NA/20 × Kort M27 objective lens using identical settings (LSM 700, Zeiss, Germany). The relative fluorescence intensity of individual hair cell was measured and analyzed using ImageJ software.

### Vestibular Function Examination

Vestibular function of mice was evaluated as described previously (Li et al., 2021). Circling stereotyped movement was counted to record compulsive circles around the animal’s hips. Swimming test was performed to observe swimming behavior ranging from normal swimming to drowning. Swimming test scores were defined as follows: 0, normal swimming; 1, irregular swimming; 2, immobile floating; and 3, underwater tumbling. For rotarod test, mice were placed on the rod apparatus (HB-600, Ruanlong, China) that was set to accelerate from 0 to 50 rpm over a 3-min period. Mice were trained for seven consecutive days and the time before dropping was recorded on day 4–7. Four trials were performed on each day and the second, third and fourth trials were measured and analyzed.

### Statistical Analysis

All experiments were performed at least three times independently. Data were shown as means ± standard error of mean (SEM). Student’s two-tailed unpaired *t* test was used to determine statistical significance when the results show normal distribution; otherwise, Mann-Whitney *U* test is used. *P* < 0.05 was considered statistically significant.

## Results

### BAIAP2L2 Is Localized at the Tips of Shorter-Row Stereocilia in Vestibular Hair Cells

**FIGURE 1:**
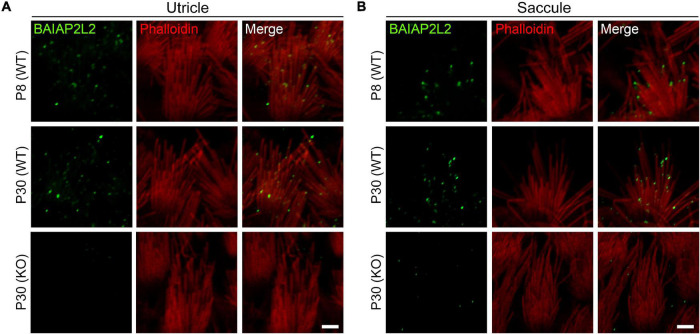
BAIAP2L2 is localized at the tips of shorter-row stereocilia in VHCs. Whole-mount immunostaining using a specific anti-BAIAP2L2 antibody (green) was performed to examine the localization of BAIAP2L2 in the stereocilia of utricular **(A)** and saccular **(B)** hair cells. Steoreociliary F-actin core was visualized using TRITC-conjugated phalloidin (red). The genotypes and ages of mice are indicated. Scale bar, 2 μm.

We first examined the localization of BAIAP2L2 in the VHC stereocilia by performing whole-mount immunostaining and confocal microscopy using a specific anti-BAIAP2L2 antibody. The stereociliary F-actin core was visualized by TRITC-conjugated phalloidin. The results reveal that BAIAP2L2 immunoreactivity is localized at the tips of utricular hair cell stereocilia at postnatal day 8 (P8) (Figure 1A and Supplementary Figure 1A, top panel). Noticeably, BAIAP2L2 immunoreactivity is more enriched at the tips of the shorter-row stereocilia (Figure 1A, top panel). Similar results were obtained in P30 utricular hair cells (Figure 1A and Supplementary Figure 1A, middle panel). No BAIAP2L2 immunoreactivity is detected in the homozygous *Baiap2l2* knockout mice, confirming the specificity of the antibody (Figure 1A and Supplementary Figure 1A, bottom panel). Similar expression pattern was also observed in saccular hair cells (Figure 1B and Supplementary Figure 1B).

### Loss of BAIAP2L2 Does Not Affect the Development or Maintenance of Vestibular Hair Cell Stereocilia

**FIGURE 2:**
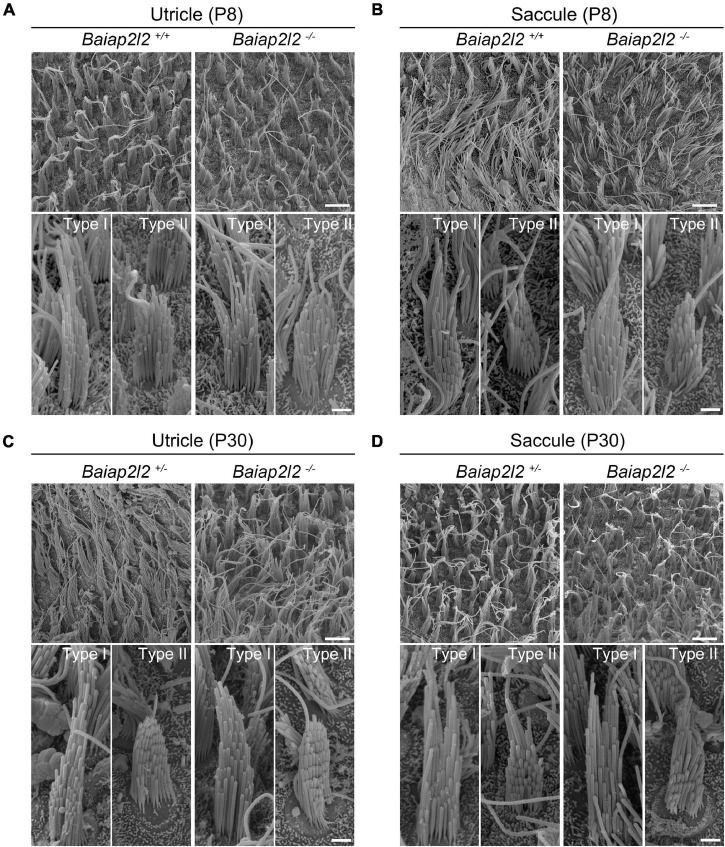
Stereocilia morphology is unaffected in VHCs of *Baiap2l2* knockout mice. SEM was performed to examine the stereocilia morphology of P8 utricle **(A)**, P8 saccule **(B)**, P30 utricle **(C)**, and P30 saccule **(D)** in mice of different genotypes as indicated. In each panel, low-magnification images and high magnification images of type I and II VHCs are shown at the top and bottom, respectively. Scale bar, 5 μm (in low-magnification images) and 1 μm (in high-magnification images).

Phalloidin staining reveals largely unaffected stereocilia morphology in *Baiap2l2^–/–^* VHCs (Figures 1A,B). Scanning electron microscopy (SEM) was then employed to further examine the morphology of VHC stereocilia in *Baiap2l2* knockout mice. When examined at P8, the morphology of both utricular and saccular VHC stereocilia in *Baiap2l2^–/–^* mice is indistinguishable from that in control mice (Figures 2A,B). Similar results were observed in *Baiap2l2^–/–^* mice at P30 (Figures 2C,D). Taken together, our present data suggest that loss of BAIAP2L2 does not affect the development or maintenance of VHC stereocilia.

### Loss of BAIAP2L2 Does Not Affect the Mechano-Electrical Transduction Function of Vestibular Hair Cells

**FIGURE 3:**
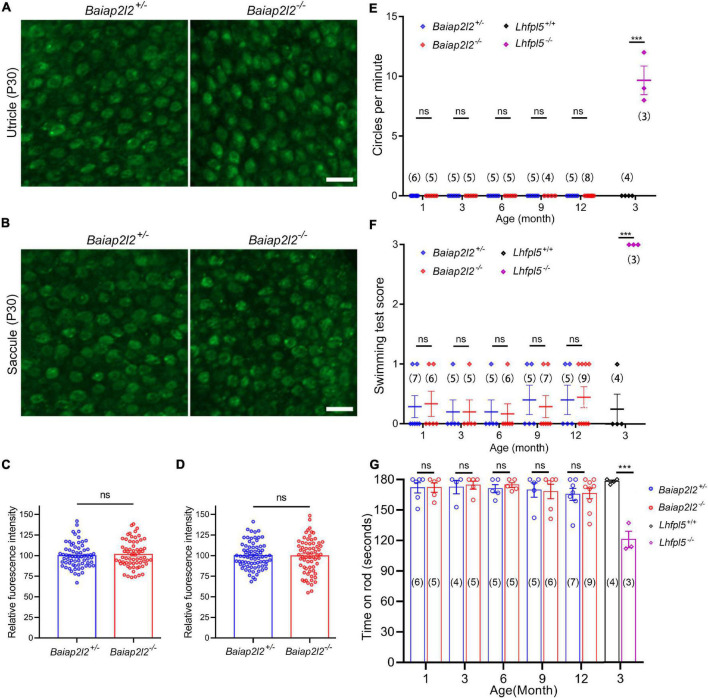
Vestibular function is unaffected in *Baiap2l2* knockout mice. **(A,B)** FM1-43FX uptake by P30 utricular **(A)** and saccular **(B)** hair cells from *Baiap2l2^+/–^* or *Baiap2l2^–/–^* mice was examined using confocal microscope. Scale bars, 10 μm. **(C,D)** FM1-43FX uptake was quantified according to the results from **(A,B)**, respectively. **(E–G)** The vestibular function of *Baiap2l2^+/–^* or *Baiap2l2^–/–^* mice of different ages was evaluated by examining circling stereotyped movement **(E)**, swimming test **(F)**, and rotarod test **(G)**. *Lhfpl5^–/–^* mice were included as positive control. Numbers of animals in each group are indicated in brackets. ns, not significant; ***p < 0.001.

The unaffected stereocilia morphology suggests that MET function might be normal in *Baiap2l2^–/–^* VHCs. We then examined the MET function of *Baiap2l2^–/–^* VHCs by performing FM 1-43FX uptake experiment. FM 1-43FX is a fixable fluorescent dye that could enter hair cells through MET channels when applied briefly, therefore is often used as an indicator of hair cell MET function (Gale et al., 2001; Meyers et al., 2003). The results show that FM 1-43FX dye uptake of utricular VHCs in *Baiap2l2^–/–^* mice at P8 is comparable to that in control mice (Supplementary Figures 2A,B). Similar results were obtained in P8 saccular VHCs (Supplementary Figures 2C,D). We then examined the MET function of adult VHCs at P30 by performing FM 1-43FX uptake experiment, which also did not reveal any difference between *Baiap2l2^–/–^* and control mice (Figures 3A–D). Therefore, our present data suggest that the MET function of VHCs is not affected by loss of BAIAP2L2.

### Loss of BAIAP2L2 Does Not Affect Vestibular Function in Mice

We then moved on to examine the vestibular function of *Baiap2l2^–/–^* mice. Mutation in *Lhfpl5* gene, which encodes for MET component LHFPL5, leads to deafness and balance dysfunction (Longo-Guess et al., 2005). Therefore, *Lhfpl5^–/–^* mice were included in the present experiments as positive control. Consistent with the previous report, *Lhfpl5^–/–^* mice show typical circling stereotyped movement, suggesting of balance dysfunction (Figure 3E). However, *Baiap2l2^–/–^* mice at ages of up to 12 months do not show any circling stereotyped movement (Figure 3E).

The vestibular function of *Baiap2l2^–/–^* mice was further evaluated by swimming test and rotarod test. In both tests, *Baiap2l2^–/–^* mice at ages of up to 12 months perform indistinguishably from control *Baiap2l2^+/–^* mice (Figures 3F,G). In contrast, *Lhfpl5^–/–^* mice show abnormal swimming behavior and are easier to fall off the rotarod (Figures 3F,G). Taken together, our present data suggest that the vestibular function is not affected by loss of BAIAP2L2.

### Loss of BAIAP2L2 Does Not Affect the Stereociliary Tip Localization of CAPZB2 or EPS8 in Vestibular Hair Cells

**FIGURE 4:**
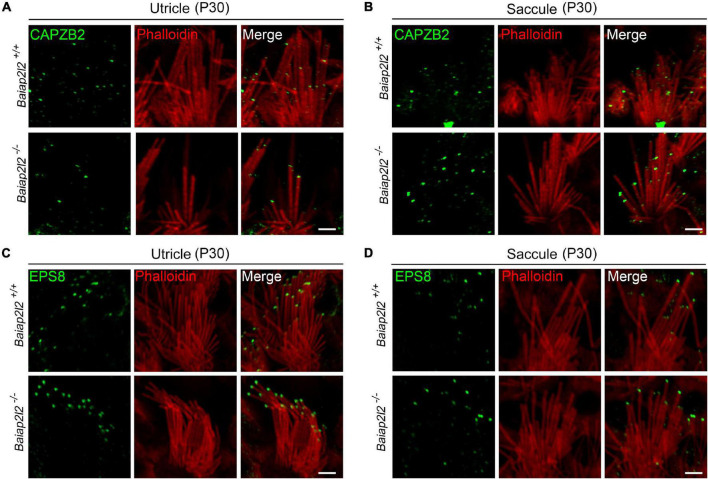
Stereociliary tip localization of CAPZB2 and EPS8 is unaffected in VHCs of *Baiap2l2* knockout mice. Whole-mount immunostaining and confocal microscopy were performed to examine the localization of CAPZB2 in utricular **(A)** and saccular **(B)** hair cells of P30 *Baiap2l2*^+/+^ or *Baiap2l2^–/–^* mice. Similar experiments were performed to examine the localization of EPS8 in utricular **(C)** and saccular **(D)** hair cells of P30 *Baiap2l2*^+/+^ or *Baiap2l2^–/–^* mice. TRITC-conjugated phalloidin was used to visualize stereociliary F-actin core. Scale bars, 2 μm.

To explore the possible reason why loss of BAIAP2L2 does not affect VHC stereocilia or vestibular function, we tried to examine the stereociliary localization of other row 2 complex components in VHCs by performing whole-mount immunostaining and confocal microscopy. Here, we focused on CAPZB2 since its stereociliary tip localization in cochlear hair cells has been shown to depend on BAIAP2L2 (Yan et al., 2021). The results show that in the utricle of P30 control mice, CAPZB2 immunoreactivity is localized at the tips of shorter-row stereocilia as reported previously (Avenarius et al., 2017; Figure 4A, top panel). Interestingly, CAPZB2 immunoreactivity is unaffected in the utricle of *Baiap2l2^–/–^* mice (Figure 4A, bottom panel), which is consistent with the normal development and maintenance of VHC stereocilia in *Baiap2l2^–/–^* mice. Similar results were observed in the saccule (Figure 4B).

EPS8 is a row 1 complex component and is responsible for the stereociliary tip localization of BAIAP2L2 in cochlear hair cells (Carlton et al., 2021). Our results show that EPS8 immunoreactivity is localized at the tips of the taller-row stereocilia in the utricle or saccule of both *Baiap2l2*^+/+^ and *Baiap2l2^–/–^* mice (Figures 4C,D). Taken together, the present data show that row 1 and row 2 complex are largely unaffected in the VHC stereocilia of *Baiap2l2^–/–^* mice.

## Discussion

BAIAP2L2 is a recently identified row 2 complex component that localizes at the tips of shorter-row mechanotransducing stereocilia in cochlear hair cells (Carlton et al., 2021; Yan et al., 2021). BAIAP2L2 inactivation results in mechanotransducing stereocilia degeneration in cochlear hair cells, and leads to profound hearing loss (Carlton et al., 2021; Yan et al., 2021). In the present work, we show that BAIAP2L2 is also localized at the tips of shorter-row stereocilia in VHCs. Unexpectedly, our data reveal that BAIAP2L2 inactivation does not affect the development/maintenance of VHC stereocilia as well as vestibular function.

There are evidences suggesting that deficiency of row 2 complex components might affect cochlear and vestibular hair cells differently. For example, EPS8L2 or MYO15A-L inactivation results in degeneration of the mechanotransducing stereocilia in cochlear hair cells, but does not significantly affect vestibular function and/or VHC stereocilia morphology (Furness et al., 2013; Fang et al., 2015). ESP8L2 localizes at the tips of most VHC stereocilia including the taller ones (Furness et al., 2013; Avenarius et al., 2017). Meanwhile, row 1 complex component EPS8 is mostly enriched at the tips of taller-row VHC stereocilia, raising the possibility that row 1 and 2 complex components might work cooperatively in VHC stereocilia and compensate for the loss of each other (Furness et al., 2013; Avenarius et al., 2017). Our present results suggest that similar functional compensation might also happen in *Baiap2l2* knockout mice, which awaits further investigations.

One of the candidates responsible for this possible compensation is its homolog BAIAP2L1 (also known as IRTKS), whose expression has been detected in the hair cells through transcriptome studies (umgear.org). In the gut, BAIAP2L1 could localize EPS8 to the developing microvilli of the brush border and regulate their growth (Postema et al., 2018). Examination of the precise localization of BAIAP2L1 in the stereocilia of cochlear and vestibular hair cells, and analysis of *Baiap2l1* knockout mice and *Baiap2l1*/*Baiap2l2* double knockout mice will help to address this question.

Similarly to BAIAP2L2, CAPZB2 and TWF2 are more enriched at the tips of shorter-row VHC stereocilia (Avenarius et al., 2017). CAPZB2 is a capping protein that binds to the barbed ends of F-actin and prevents both actin polymerization and depolymerization (Caldwell et al., 1989). CAPZB2 functions as a heterodimer formed together with CAPZA1/2, both of which are detected at the tips of stereocilia (Shin et al., 2013; Avenarius et al., 2017). In sharp contrast to BAIAP2L2/EPS8L2/MYO15A-L, CAPZB2 inactivation leads to VHC stereocilia deficits as well as compromised vestibular function (Avenarius et al., 2017). *Capzb2*-deficient VHC stereocilia are normal when examined at P2, but become severely disrupted at P7-P9 in some VHCs (Avenarius et al., 2017). A common phenotype in *Capzb2*-deficient VHCs at P7-P9 is missing of the shortest stereocilia, with the intermediate or highest stereocilia largely unaffected (Avenarius et al., 2017). Consistent with the important role of CAPZB2 in VHC stereocilia, the stereociliary tip localization of CAPZB2 is unaffected in *Baiap2l2*-deficient VHCs. In contrast, in the cochlear hair cells, the stereociliary tip localization of CAPZB2 is dependent on functional BAIAP2L2 (Yan et al., 2021). The different dependency of CAPZB2 localization on BAIAI2L2 in cochlear and vestibular hair cells might explain the different auditory and balancing phenotypes in *Baiap2l2* knockout mice. Detailed examination of the localization of row 2 proteins in VHC stereocilia using super-resolution microscopy might help to learn more about the underlying mechanism (Liu et al., 2019; Qi et al., 2019, 2020).

## Data Availability Statement

The original contributions presented in the study are included in the article/Supplementary Material, further inquiries can be directed to the corresponding authors.

## Ethics Statement

The animal study was reviewed and approved by the Animal Ethics Committee of Shandong University School of Life Sciences.

## Author Contributions

WZ and ZX: study concept and design. KY, CQ, YW, and WZ: acquisition of data. KY, CQ, YW, WZ, and ZX: analysis and interpretation of data. KY, WZ, and ZX: drafting the manuscript. All authors contributed to the article and approved the submitted version.

## Conflict of Interest

The authors declare that the research was conducted in the absence of any commercial or financial relationships that could be construed as a potential conflict of interest.

## Publisher’s Note

All claims expressed in this article are solely those of the authors and do not necessarily represent those of their affiliated organizations, or those of the publisher, the editors and the reviewers. Any product that may be evaluated in this article, or claim that may be made by its manufacturer, is not guaranteed or endorsed by the publisher.
