# Supplementary material for: BAIAP2L2 Inactivation Does Not Affect Stereocilia Development or Maintenance in Vestibular Hair Cells
Source: Front Mol Neurosci. 2022 Feb 15;15:829204. doi: 10.3389/fnmol.2022.829204 (PMC8886116; doi:10.3389/fnmol.2022.829204)
Supplement: Supplementary file 1 [file Data_Sheet_1.PDF]

BAIAP2L2 inactivation does not affect stereocilia development or maintenance in vestibular hair cells

Keji Yan[1], Chengli Qu[1], Yanfei Wang[1], Wen Zong[2*], Zhigang Xu[1,3*]

[1]Shandong Provincial Key Laboratory of Animal Cell and Developmental Biology, School of Life Sciences, Shandong University, Qingdao, Shandong 266237, China

[2]State Key Laboratory of Microbial Technology, Shandong University, Qingdao, Shandong 266237, China

[3]Shandong Provincial Collaborative Innovation Center of Cell Biology, Shandong Normal University, Jinan, Shandong 250014, China

[*]Correspondence

Wen Zong, State Key Laboratory of Microbial Technology, Shandong University, Qingdao, Shandong 266237, China. E-mail: wenzong@sdu.edu.cn (W. Z.)

Zhigang Xu, Shandong Provincial Key Laboratory of Animal Cell and Developmental Biology, School of Life Sciences, Shandong University, Qingdao, Shandong 266237, China. E-mail: xuzg@sdu.edu.cn (Z. X.)

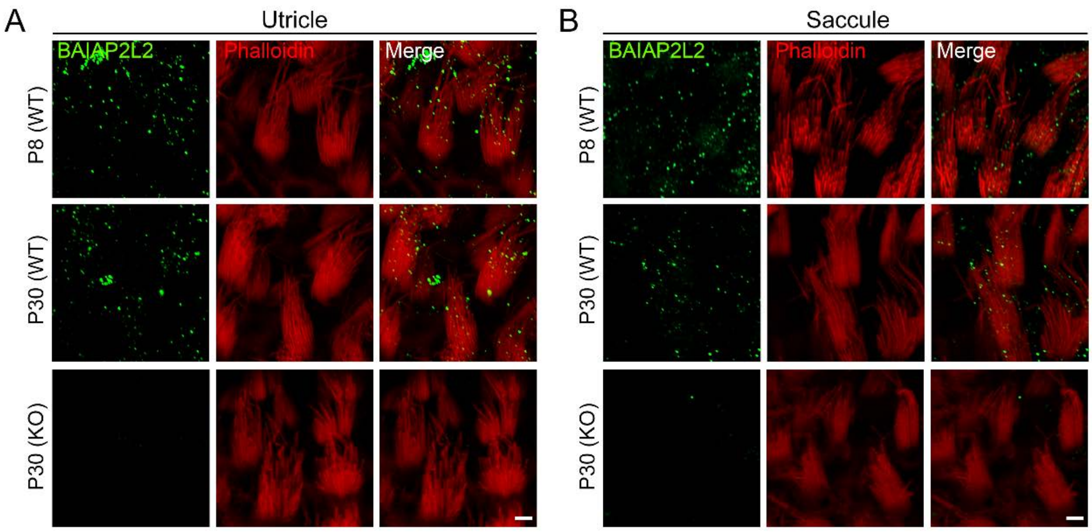

Supplementary Figure 1. BAIAP2L2 is localized at the tips of stereocilia in VHCs. Whole-mount immunostaining using a specific anti-BAIAP2L2 antibody (green) was performed to examine the localization of BAIAP2L2 in the stereocilia of utricular (A) and saccular (B) hair cells. Steoreociliary F-actin core was visualized using TRITC-conjugated phalloidin (red). The genotypes and ages of mice are indicated. Scale bar, 2 μm.

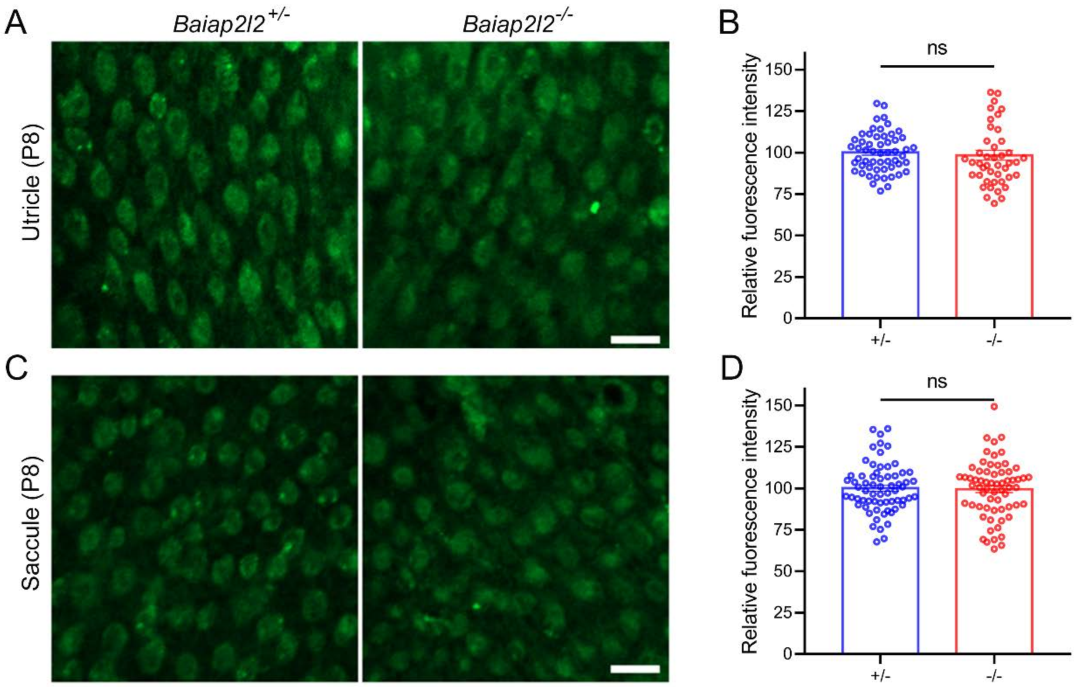

Supplementary Figure 2. MET function is unaffected in VHCs of P8 *Baiap2l2* knockout mice. MET function was evaluated by performing FM1-43FX uptake experiments in P8 utricular (A) and saccular (C) hair cells of *Baiap2l2*$^{+/-}$ or *Baiap2l2*$^{-/-}$ mice. Images were taken using a confocal microscope. Scale bars, 10 µm. (B) and (D) FM1-43FX uptake was quantified according to the results from (A) and (C), respectively. ns, not significant.
